# A clique-based method for the edit distance between unordered trees and its application to analysis of glycan structures

**DOI:** 10.1186/1471-2105-12-S1-S13

**Published:** 2011-02-15

**Authors:** Daiji Fukagawa, Takeyuki Tamura, Atsuhiro Takasu, Etsuji Tomita, Tatsuya Akutsu

**Affiliations:** 1 Faculty of Culture and Information Science, Doshisha University, Kyoto 610-0394, Japan; 2 Bioinformatics Center, Institute for Chemical Research, Kyoto University, Kyoto 611-0011, Japan; 3 National Institute of Informatics, Tokyo 101-8430, Japan; 4 University of Electro-Communications, Tokyo 182-8585, Japan; 5 Research and Development Initiative, Chuo University, Tokyo 112-8551, Japan

## Abstract

**Background:**

Measuring similarities between tree structured data is important for analysis of RNA secondary structures, phylogenetic trees, glycan structures, and vascular trees. The edit distance is one of the most widely used measures for comparison of tree structured data. However, it is known that computation of the edit distance for rooted unordered trees is NP-hard. Furthermore, there is almost no available software tool that can compute the exact edit distance for unordered trees.

**Results:**

In this paper, we present a practical method for computing the edit distance between rooted unordered trees. In this method, the edit distance problem for unordered trees is transformed into the maximum clique problem and then efficient solvers for the maximum clique problem are applied. We applied the proposed method to similar structure search for glycan structures. The result suggests that our proposed method can efficiently compute the edit distance for moderate size unordered trees. It also suggests that the proposed method has the accuracy comparative to those by the edit distance for ordered trees and by an existing method for glycan search.

**Conclusions:**

The proposed method is simple but useful for computation of the edit distance between unordered trees. The object code is available upon request.

## Background

Analysis of tree structured data is important in bioinformatics because there exist various kinds of tree structured biological data, which include RNA secondary structures [1,2], phylogenetic trees [3-5], glycans (i.e., sugar chains) [6-9], and vascular trees [10,11]. Various techniques have been applied to analyses of these tree structured data. Though machine learning techniques have been extensively applied to analysis of glycan structures [7-9], it is still important to develop simple comparison/search methods because machine learning methods are not appropriate for fast search of similar objects. Indeed, in analysis of biological sequences, such sequence search/comparison tools as FASTA, BLAST and SSEAECH are still widely used. Therefore, it is worthy to develop search/comparison methods for tree structured data. In order to compare tree structured data, it is required to define some measure of similarity or dissimilarity between two trees. Among various measures, the *tree edit distance* is the most fundamental and has been extensively studied [12]. It measures the distance between two trees by means of the minimum cost sequence of edit operations that transforms one tree into another tree, where an edit operation is either a *deletion* of a node, an *insertion* of a node, or a *substitution* of a label of a node. For the tree edit distance problem for ordered trees, Tai developed an *O*(*n*^6^) time algorithm [13], where *n* is the number of nodes in a larger input tree. Several improvements followed from this work. Demaine *et al.* recently developed an *O*(*n*^3^) time algorithm and showed that this bound is optimal under some computation strategy [14].

The tree edit distance between ordered trees is useful if the ordering among children has an important meaning (e.g., for RNA secondary structures). However, in some applications, it is preferable to regard input trees as unordered trees. At least, in many applications, more flexible matching can be made possible if input trees are regarded as unordered trees and thus the chance that similar data is missed can be decreased. It is to be noted that edit distance for unordered trees is always smaller than that for ordered trees. Unfortunately, Zhang *et al.* proved that the tree edit distance problem for unordered trees is NP-hard [15]. Furthermore, Zhang and Jiang proved that it is MAX SNP-hard [16], which means that there exists no polynomial time approximation scheme unless P=NP. In order to cope with this hardness, Akutsu et al. developed a fixed parameter algorithm which works in *O*(2.62*^k^* · *poly*(*n*)) time [17], where *k* is the maximum allowed edit distance. Their algorithm might be useful for comparison of very similar trees (i.e., *k* is small). However, it is not useful for comparison of non-similar trees. Horesh et al. developed an A* algorithm [3]. Their algorithm works efficiently for moderate size trees. However, their algorithm can only handle unit cost cases (i.e., the cost of each edit operation is 1). Some alternatives to the tree edit distance for unordered trees have been proposed [6,12,18,19]. However, none of them is widely accepted as a measure of similarity for unordered trees. Therefore, it is still needed to develop a practical method for calculating tree edit distance between unordered trees.

In this paper, we propose a practical method using algorithms for computing the *maximum clique*. The idea of the method is simple: the edit distance problem is reduced to the maximum clique problem and then practical solvers for the maximum clique problem are applied. The maximum clique problem is a fundamental problem in computer science and is to find a complete subgraph of the maximum number of vertices in a given undirected graph. Though the maximum clique problem is proven to be NP-hard, several practical algorithms have been developed and successfully applied for practical problems [20-23]. By utilizing such algorithms [20,21], we can solve the edit distance problem for unordered trees of moderate size (i.e., trees with 30 ~ 45 nodes). Though similar reductions have been proposed for similar edit distance problems [24,25], to our knowledge, it is the first method that *exactly* solves the proper tree edit distance problem for unordered trees using maximum clique, where we use the fastest maximum clique algorithms [21,22] developed by one of the authors and his collaborators. Furthermore, to our knowledge, it is the first practical method for computing the unordered tree edit distance with general editing cost functions.

In order to evaluate the proposed method, we perform computational experiments using glycan structure data stored in the KEGG database [26]. The result suggests that our proposed method can efficiently compute the edit distance for moderate size unordered trees. It also suggests that the proposed method has the accuracy comparative to those by the edit distance for ordered trees and by an existing method for glycan search.

## Methods

### Tree edit distance

**Figure 1:**
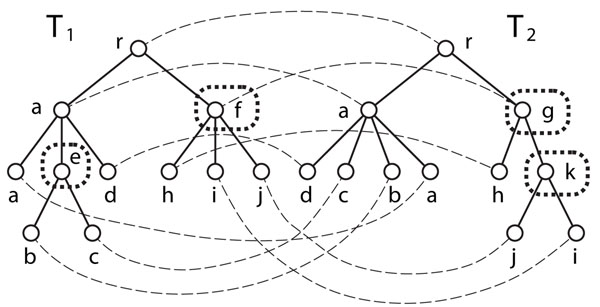
**Example of tree edit operations and edit distance mapping under the unit cost model. ***T*_2_ is obtained from *T*_1_ by deletion of node (labeled with) e, insertion of node k and substitution of node f. The corresponding mapping *M* is shown by broken curves.

Here, we briefly review *tree edit distance* and *edit distance mapping* (see also Figure 1) for rooted, labelled and unordered trees [12,15,16].

Let *T* be a rooted unordered tree. We assume that each node *v* has a label *ℓ*(*v*) over an alphabet Σ. *r*(*T*), *V*(*T*), and *E*(*T*) denote the root of *T*, the set of nodes in *T*, and the set of edges in *T*, respectively. For a node *v ∈ V*(*T*), *anc*(*v*) denotes the set of ancestors of *v*. In the following, *n* denotes the number of nodes in a larger input tree (i.e., *n* = max{|*V*(*T*_1_)|, |*V*(*T*_2_)|}).

An *edit operation on a tree T* is either a *deletion*, an *insertion*, or a *substitution*, where each operation is defined as follows (see also Figure 1):

**Deletion:** Delete a non-root node *v* in *T* with parent *u*, making the children of *v* become children of *u*. The children are inserted in the place of *v* into the set of the children of *u*.

**Insertion:** Inverse of delete. Insert a node *v* as a child of *u* in *T*, making *v* the parent of some of the children of *u*.

**Substitution:** Change the label of a node *v* in *T*.

For each of edit operations, the *cost* is defined as follows:

*γ*(*a, b*): cost of substituting a node with label *a* to label *b*,

*γ*(*a, ∈*): cost of deleting a node labeled with *a*,

*γ*(*∈, a*): cost of inserting a node labeled with *a*.

The *edit distance dist*(*T*_1_*, T*_2_) between two unordered trees *T*_1_ and *T*_2_ is defined as the cost of the minimum cost sequence of edit operations that transforms *T*_1_ to *T*_2_. In this paper, we adopt the following standard assumption so that *dist*(*T*_1_, *T*_2_) becomes a distance metric [12,15]:

• *γ*(*a, b*) ≥ 0 for any (*a, b*) *∈* Σ′ × Σ′,

• *γ*(*a, a*) = 0 for any *a ∈* Σ′,

• *γ*(*a, b*) = *γ*(*b, a*) for any (*a, b*) ∈ Σ′ × Σ′,

• *γ*(*a, c*) ≤ *γ*(*a, b*) + *γ*(*b, c*) for any *a,b,c ∈* Σ′ × Σ′ × Σ′,

where Σ′ = ΣU {∈}. We call *T*_2_ a *subtree* of *T*_1_ if *T*_2_ is obtained from *T*_1_ only by deletion operations. It should be noted that this definition of subtree is different from a subgraph of a tree.

There exists a close relationship between the edit distance and the *edit distance mapping* (or just *mapping)*[12,15]. *M ⊆ V* (*T*_1_) × *V*(*T*_2_) is called a *mapping* if the following conditions are satisfied for any two pairs (*u*_1_, *v*_1_), (*u*_2_, *v*_2_) ∈ *M*:

(i) *u*_1_ = *u*_2_ iff *v*_1_ = *v*_2_,

(ii) *u*_1_ ∈ *anc*(*u*_2_) iff *v*_1_ ∈ *anc*(*v*_2_).

Let *I*_1_ and *I*_2_ be the sets of nodes in *V*(*T*_1_) and *V*(*T*_2_) not appearing in *M*, respectively. Then, the following relation holds [12,15]:

Here we define a *score function f*(*u, v*) for (*u, v*) *∈ V*(*T*_1_) × *V*(*T*_2_) by

*f*(*u, v*) = *γ*(*ℓ*(*u*), *∈*) + *γ*(*∈, ℓ*(*v*)) - *γ*(*ℓ*(*u*), *ℓ*(*v*)).

It is seen that *f*(*u, v*) = *f*(*v, u*) ≥ 0 holds. It should also be noted that under the unit cost model (i.e., *γ*(*a, b*) = 1 for all *a ≠ b*), *f*(*v, v*) = 2 and *f*(*u, v*) = 1 hold for *ℓ*(*u*) ≠ *ℓ*(*v*). Let *score*(*M*) be the score of a mapping *M* defined by *score*(*M*) = *∑*_(_*_u,v_*_)∈_*_M_ f*(*u, v*). Let *M_OPT_* be the mapping with the maximum score. Then, we can see from the definition that the following property holds [17]:(1)

assuming that the root of *T*_1_ corresponds to the root of *T*_2_ in *M_OPT_*, where this assumption can be removed if we add dummy nodes having the same label to *T*_1_ and *T*_2_ as the new roots.

### Reduction to maximum clique

Let *G*(*V, E*) be an undirected graph. Then, a subgraph *G′*(*V′, E′*) of *G*(*V, E*) is called a *clique* if it is a complete subgraph (i.e., {{*v_i_,v_j_*} | *v_i_, v_j_ ∈ V′, v_i_* ≠ *v_j_* } = *E′*). The *maximum clique* problem is to find a maximum clique (i.e., a clique with the maximum number of vertices) in a given undirected graph. Though the maximum clique problem is known to be NP-hard, several practical algorithms have been developed [20-23]. In some cases, weighted versions of the maximum clique problem are utilized. Among such variants, we consider the case that weights are given to vertices. Let *w*(*v*) denote the weight of a vertex *v* in *G*(*V, E*). Then, a weighted version of the maximum clique problem is to find a clique *G′*(*V′, E′*) which maximizes *∑_v∈V′_w*(*v*). In this paper, we call this variant *the maximum vertex weighted clique problem*, whereas the maximum clique problem denotes the original one.

Our proposed method is based on a simple reduction from the edit distance problem for unordered trees to the maximum clique problem. Based on Eq. (1), for calculating the tree edit distance, it is enough to find a mapping *M* which maximizes *∑*_(*u, v*)*∈M*_*f*(*u, v*). In order to find such a mapping, we construct an undirected graph *G*(*V,E*) from two input trees *T*_1_ and *T*_2_ by

*V* = {(*u, v*) | *u ∈V*(*T*_1_)*, u* ≠ *r* (*T*_1_)*, v ∈V*(*T*_2_)*, v* ≠ *r*(*T*_2_)}*,*

*E* = {{(*u*_1_*,v*_1_)*,* (*u*_2_*,v*_2_ )} | *u*_1_ ≠ *u*_2_*, v*_1_ ≠ *v*_2_*, u*_1_*∈anc*(*u*_2_) iff *v*_1_ ∈ *anc*(*v*_2_)*, u*_2_ ∈ *anc*(*u*_1_) iff *v*_2_ ∈ *anc*(*v*_1_)},

where the first two conditions and the last two conditions in the definition of *E* correspond to conditions (i) and (ii) for the edit distance mapping, respectively. We can see that there is a one-to-one correspondence between the set of cliques and the set of edit distance mappings, where we let *r*(*T*_1_) correspond to *r*(*T*_2_) (because the root cannot be deleted or inserted). Here, we assign weight *w*(*x*) to each vertex *x* = (*u, v*) *∈V* by *w*(*x*) *= f*(*u, v*). Then, we can see from Eq. (1) that the tree edit distance can be obtained by finding a maximum vertex weighted clique.

**Figure 2:**
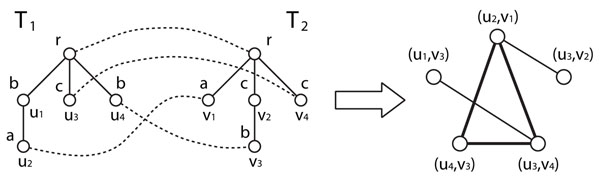
**Example of the reduction from tree edit distance to maximum clique**. We consider the case of *γ*(*a, ∈*) = *γ*(∈*, a*) *=* 1, *γ*(*a, a*) *=* 0, and *γ*(*a, b*) = 2 for *a ≠ b* (i.e., *f*(*v, v*) = 2 and *f*(*u, v*) *=* 0 for *ℓ*(*u*) *≠ℓ*(*v*)). In the left figure, both label and node ID are shown above and below parts of each node, respectively. Vertices with *f*(*u_i_, v_j_*) = 0 are omitted in the right figure. The maximum clique shown by bold lines in the right figure corresponds to the optimal edit distance mapping shown by broken lines in the left figure.

It is to be noted that if we consider the case of *γ*(*a, ∈*) = *γ*(∈*, a*) = 1, *γ*(*a, a*) = 0 for all *a ∈* Σ, and *γ*(*a, b*) = 2 for all *a ≠ b*, we have *f*(*v, v*) *=* 2 and *f*(*u, v*) *=* 0 for *ℓ*(*u*) *≠ ℓ*(*v*), and thus we can use a non-weighted version of maximum clique algorithms (see Figure 2). In such a case, the resulting mapping gives a largest common subtree (a tree with the largest number of nodes which is a subtree of both *T*_1_ and *T*_2_) [17].

### Maximum clique algorithms

In this study, we use algorithms for both the maximum clique problem and the maximum vertex weighted clique problem. For both problems, Tomita and his collaborators have been developing several algorithms. Recent studies on comparison with other existing algorithms suggest that *their algorithms are fastest in most cases*[22]. Based on several preliminary experiments, we chose MCQ and MWCQ as algorithms for the maximum clique problem and the maximum vertex weighted clique problem, respectively, where MWCQ is basically an extended version of MCQ. Details of MCQ and MWCQ are given in [21] and [20], respectively.

Though there are some theoretical studies on related algorithms [23], the worst case time complexities of MCQ and MWCQ are left open. Therefore, we cannot discuss the time complexity of our proposed method, whereas it is straight-forward to see that the graph obtained by reduction from input trees has *O*(|*V*(*T*_1_)| × |*V*(*T*_2_)|) nodes and *O*(|*V*(*T*_1_)|^2^ × |*V*(*T*_2_)|^2^) edges.

## Results

We implemented the above mentioned maximum clique-based method (MCQ-based method) and maximum vertex weighted clique-based method (MWCQ-based method) using C language. We performed computational experiments using a PC with Intel Core 2 Duo 2.8 GHz CPU and 3.48 GB RAM running under the Cygwin/Widnows XP operating system. As tree structures, we used glycan structures obtained from KEGG/Glycan database [26].

### Results on efficiency

First we examined the computational efficiency of MWCQ-based method, where we used the standard weighting scheme (i.e., *f*(*v, v*) = 2 and *f*(*u, v*) = 1 for *ℓ*(*u*) *≠ ℓ*(*v*)) corresponding to the unit cost edit distance. We randomly selected 100 pairs of glycan structures with a specified range of the total number of nodes (i.e., the sum of the numbers of nodes in *T*_1_ and *T*_2_) and measured the average CPU time (user time) per pair. Unbalanced cases in which the size of one structure was not larger than 1/3 of the other structure were excluded. For each of the ranges in 60 ~ 79, we took the average over 20 pairs because there did not exist an enough number of pairs, where we could use 18 pairs among 20 pairs for the range of 65 ~ 69 because there were two very bad cases for which the program could not output a solution within 10 minutes. For the ranges of 80 ~ 84 and 85 ~ 89, we could use only 9 and 5 pairs, respectively. The result is shown in Table 1. From this table, it is seen that the proposed method works efficiently for moderate size trees (i.e., trees with 30 ~ 45 nodes), which means that the proposed method works efficiently for most glycan structures.

**Table 1:** CPU time on maximum vertex weighted clique-based method

total number of nodes	average CPU time (sec.)
30 ~ 34	0.004340
35 ~ 39	0.004990
40 ~ 44	0.015200
45 ~ 49	0.050800
50 ~ 54	0.473000
55 ~ 59	2.160000
60 ~ 64	3.020000
65 ~ 69	15.300000
70 ~ 74	4.380000
75 ~ 79	2.610000
80 ~ 84	7.930000
85 ~ 89	232.000000

Next we examined the computational efficiency of MCQ-based method, which corresponds to the case of computation of the largest common subtree (i.e., *f*(*v, v*) = 2 and *f*(*u, v*) = 0 for *ℓ*(*u*) *≠ ℓ*(*v*)). As in the case of MWCQ-based method, we randomly selected 100 pairs of glycan structures with a specified range of the total number of nodes and measured the average CPU time, where we used a fewer number of pairs when the number of nodes was no less than 60 as in the case of MWCQ-based method. The result is shown in Table 2. From this table, it is seen that MCQ-based method works very fast for most glycan structures. It is to be noted that CPU time does not necessarily increase as the size of input trees because the size of transformed clique instances strongly depends on the distribution of identical labels in input trees and thus does not so much depend on the size of input trees.

**Table 2:** CPU time on maximum clique-based method

total number of nodes	average CPU time (sec.)
30 ~ 34	0.010400
35 ~ 39	0.000191
40 ~ 44	0.000203
45 ~ 49	0.001100
50 ~ 54	0.000780
55 ~ 59	0.004530
60 ~ 64	0.125000
65 ~ 69	4.600000
70 ~ 75	0.016400
75 ~ 79	0.032800
80 ~ 84	0.000087
85 ~ 89	0.000032

Though MCQ-based method is very fast, it makes extensive use of identity of node labels (node pairs without non-identical labels are ignored and thus the number of remaining nodes in *G*(*V, E*) becomes very small). On the other hand, MWCQ-based method takes all node pairs between *T*_1_ and *T*_2_ into account and thus is not very fast. Compared with an existing method [3], MCQ-based method is much faster but solves an easier problem (it seems from their results that their method can be applied to comparison of trees with up to 90 nodes (sum of two input trees) though CPU time is not shown in [3]). On the other hand, it seems from Table 1 that MWCQ-based method has a similar performance with that in [3] though [3] solves non-labeled cases whereas we solved labeled cases. However, MWCQ-based method has a merit: it can handle general editing cost functions whereas the method in [3] can only handle the unit editing cost.

### Results on similar structure search

Though the ordered and unordered tree edit distances are widely-accepted (dis)similarity measures on trees, we performed computational experiments in order to examine how it is useful for similarity search for glycans. We used a dataset compiled by Yamanishi et al. [9] on four properties on glycans, where we used 355 structures among 356 glycan structures listed in their list since we could not obtain one structure. Though this dataset is prepared for evaluating machine learning methods, we applied it to evaluation of search methods. We compared the following four similarity search methods: global glycan alignment and local glycan alignment implemented in the KCaM glycan search tool (version of Sept. 2004 with the default parameters) [6], unit cost ordered tree edit distance, and unit cost unordered tree edit distance (i.e., MWCQ-based method). Glycan alignment scores were introduced for efficient comparison of glycan structures. Though it is based on tree edit distance, the deletion (and corresponding insertion) operation is simplified so that only one child and its descendants can survive if a node is deleted. Therefore, there is a possibility that similar structures are missed by glycan alignment.

**Figure 3:**
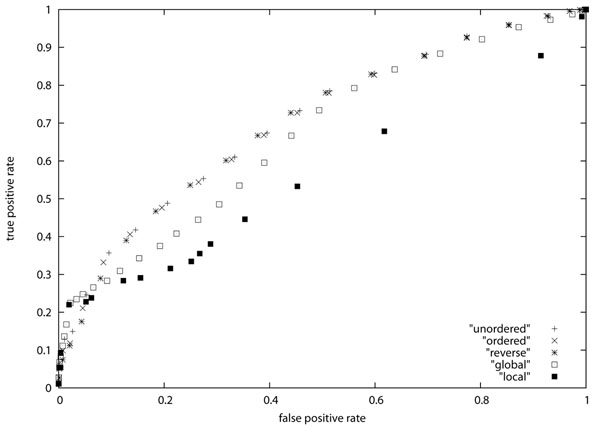
ROC curve for leukemia dataset

**Figure 4:**
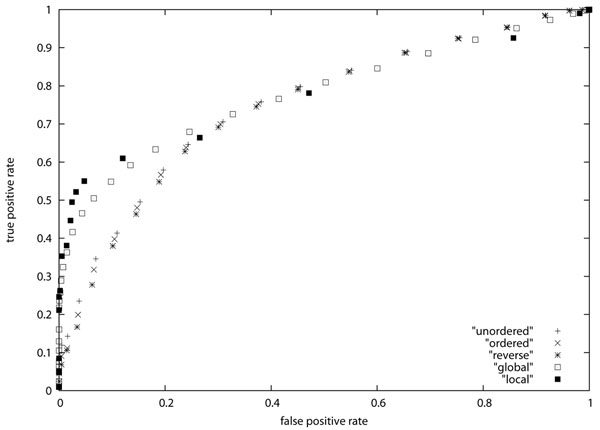
ROC curve for erythrocyte dataset

We evaluated the performance of similarity search using the AUC score [27]. In order to apply the AUC score, we need positive and negative samples. For that purpose, each pair of sequences in the dataset is regarded as a *positive sample* if the distance (resp., alignment score) is smaller (resp., greater) than a given threshold. Otherwise, it is regarded as a *negative sample*. Each positive sample is classified into either *true positive* or *false positive* according to whether sequences in the pair belong to the same class. Similarly, each negative sample is classified into either *false negative* or *true negative* according to whether sequences in the pair belong to the same class. Then, *true positive rate* and *false positive rate* are defined as the ratio of the number of true positive samples to the number of true positive and false negative samples and the ratio of the number of false positive samples to the number of false positive and true negative samples, respectively. The *ROC* (*Receiver Operating Characteristic*) *curve* is a graphical plot of the true positive rate vs. the false positive rate obtained by varying the threshold. The *AUC* (*Area Under Curve*) *score* is defined as the area under the ROC curve: AUC scores of 1 and 0.5 correspond to complete classification and random classification, respectively. The resulting ROC curves are shown in Figure 3 and Figure 4, and the resulting AUC scores are shown in Table 3. It should be mentioned that we could not obtain meaningful AUC scores for plasma and serum datasets (i.e., AUC scores for these data sets were less than 0.65 though the local alignment method produced better results). Since it seems that these data sets are not appropriate for simple search, Table 3 lists AUC scores only for leukemia and erythrocyte datasets. It is seen from the table that the tree edit distance measures are better than the alignment scores for leukemia data but are worse for erythrocyte data. It is also seen that the AUC scores for ordered tree edit distance are very close to the AUC scores for unordered tree edit distance. Though we cannot conclude that the unordered tree edit distance is better than other similarity measures for glycan search, it is comparative to other measures.

**Table 3:** Comparison of glycan similarity measures via AUC score

	AUC score		CPU time (sec.)

	leukemia	erythrocyte	
global alignment score [6]	0.686	0.797	10.08
local alignment score [6]	0.623	0.822	10.18
ordered tree edit distance	0.729	0.773	38.02
unordered tree edit distance	0.731	0.777	48.33

reversed ordered tree	0.730	0.769	37.92

In order to see more differences between ordered tree edit distance and unordered tree edit distance, we computed ordered tree edit distances when the order of children of every node was reversed in one of the input trees. The results are also shown in Table 3 (denoted as “reversed ordered tree”), Figure 3, and Figure 4 (denoted as ‘reverse’). For the result on the erythrocyte dataset, it is seen that the difference between ordered tree edit distance and unordered tree edit distance becomes larger (i.e., the difference increases from 0.004 to 0.008) though it is still small. Though we do not clearly understand the reason of this small difference, it might be because a single path in each glycan structure is relevant for the features studied in this paper.

The total CPU time for computing the distances (or scores) between all pairs of glycans in the dataset is also shown for each method in Table 3. Though the proposed clique-based method took more CPU time than other methods, the differences were not very large. It should be mentioned that we used a clique-based method for computing ordered tree edit distance for simplicity of implementation and thus CPU time on ordered tree edit distance would be much larger here than that by an efficient dynamic programming-based algorithm [14], but that is not relevant because CPU time for unordered tree edit distance is fast enough in Table 3.

**Figure 5:**
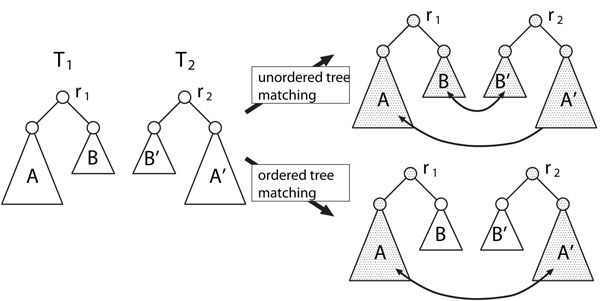
Comparison of unordered and ordered tree edit distances

**Figure 6:**
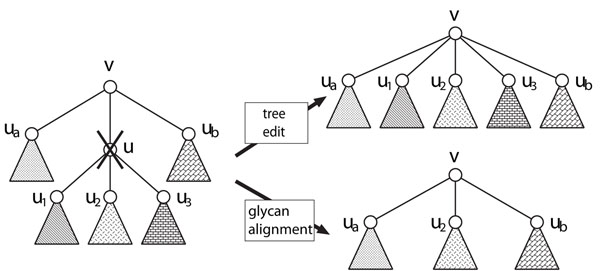
Comparison of tree edit and glycan alignment

Here, we briefly explain the methodological differences among measures. Figure 5 illustrates the difference between unordered tree edit distance and ordered tree edit distance. As shown in Figure 5, suppose that there exist two trees *T*_1_ and *T*_2_ with roots *r*_1_ and *r*_2_. Suppose further that *r*_1_ has two subtrees *A* and *B*, and *r*_2_ has two subtrees *B′* and *A′* in these orders, where *A* and *A′* (*B* and *B′*, respectively) are similar to each other, and *A* is larger than *B*. If two trees are regarded as ordered, ordered tree edit mapping takes only matching between *A* and *A′* into account. Otherwise, unordered tree mapping takes matchings between *A* and *A′*, and between *B* and *B′* into account. Figure 6 illustrates the difference of the deletion operation between tree edit and glycan alignment. In tree edit, all children of the deleted node *u* become children of the parent *v* of *u*. However, in glycan alignment, only one child can be a child of *v* and the other children are deleted along with their descendants, where the surviving child is chosen so that the resulting score is maximized. It is seen from these figures that the tree edit distance for unordered trees provides the most flexible matching.

## Conclusions

In this paper, we have proposed a clique-based method for computing the tree edit distance between rooted unordered trees. We implemented two versions: one using a maximum clique (MCQ) algorithm [21] and the other one using a maximum vertex weighted clique (MWCQ) algorithm [20].

The former one is faster than an existing A* algorithm [3]. However, it uses a non-standard editing cost scheme and thus is not more useful than the A* algorithm. The efficiency of the latter one is similar to that of the A* algorithm. However, it has two merits: it can handle general cost distances whereas the A* algorithm can only handle the unit cost distance, improvements of maximum clique algorithms lead to improvements of the efficiency of edit distance computation.

We also compared the unordered edit distance with ordered edit distance, global and local glycan alignment scores for glycan similarity search. Though the result did not show clear advantage of the unordered edit distance, it was comparative to these three measures. It is to be noted that the unit cost model was used for edit distance measures whereas score functions specialized for glycans were used for glycan alignments. Therefore, if we use editing costs specialized for glycans, we may obtain better performances. Such a development is left as future work.

Finally we again note that the edit distances for both ordered and unordered trees are already established measures for calculating the (dis)similarity between trees [12]. Therefore, application of the proposed method is not limited to glycan structures. It might be applied to analysis of various tree structure data if each tree consists of up to several tens of nodes.

## Authors' contributions

The basic idea of use of a reduction to maximum clique was born in discussions among DF, TT, AT and TA. ET provided the codes of maximum clique algorithms along with valuable comments. DF and TA implemented the method, and DF, TT and TA performed computational experiments. TA drafted the manuscript. All authors read and approved the final manuscript.

## Competing interests

The authors declare that they have no competing interests.
